# Improving the Management of Atrial Fibrillation in General Practice: Protocol for a Mixed Methods Study

**DOI:** 10.2196/21259

**Published:** 2020-11-09

**Authors:** Simon de Lusignan, F D Richard Hobbs, Harshana Liyanage, Filipa Ferreira, Manasa Tripathy, Neil Munro, Michael Feher, Mark Joy

**Affiliations:** 1 Nuffield Department of Primary Care Health Sciences University of Oxford Oxford United Kingdom; 2 Royal College of General Practitioners Research and Surveillance Centre London United Kingdom

**Keywords:** atrial fibrillation, medical record systems, computerized, general practice, cross-sectional studies, qualitative research

## Abstract

**Background:**

Atrial fibrillation (AF) is one of the commonest arrhythmias observed in general practice. The thromboembolic complications of AF include transient ischemic attack, stroke, and pulmonary embolism. Early recognition of AF can lead to early intervention with managing the risks of these complications.

**Objective:**

The primary aim of this study is to investigate if patients are managed in general practice according to current national guidelines. In addition, the study will evaluate the impact of direct oral anticoagulant use with respect to AF complications in a real-world dataset. The secondary aims of the study are to develop a dashboard that will allow monitoring the management of AF in general practice and evaluate the usability of the dashboard.

**Methods:**

The study was conducted in 2 phases. The initial phase was a quantitative analysis of routinely collected primary care data from the Oxford Royal College of General Practitioners Research and Surveillance Center (RCGP RSC) sentinel network database. AF cases from 2009 to 2019 were identified. The study investigated the impact of the use of anticoagulants on complications of AF over this time period. We used this dataset to examine how AF was managed in primary care during the last decade. The second phase involved development of an online dashboard for monitoring management of AF in general practice. We conducted a usability evaluation for the dashboard to identify usability issues and performed enhancements to improve usability.

**Results:**

We received funding for both phases in January 2019 and received approval from the RCGP RSC research committee in March 2019. We completed data extraction for phase 1 in May 2019 and completed analysis in December 2019. We completed building the AF dashboard in May 2019. We started recruiting participants for phase 1 in May 2019 and concluded data collection in July 2019. We completed data analysis for phase 2 in October 2019. The results are expected to be published in the second half of 2020. As of October 2020, the publications reporting the results are under review.

**Conclusions:**

Results of this study will provide an insight into the current trends in management of AF using real-world data from the Oxford RCGP RSC database. We anticipate that the outcomes of this study will be used to guide the development and implementation of an audit-based intervention tool to assist practitioners in identifying and managing AF in primary care.

**International Registered Report Identifier (IRRID):**

RR1-10.2196/21259

## Introduction

Atrial fibrillation (AF) is one of the commonest arrhythmias observed in clinical practice [1]. Globally, the incidence of AF is rising. The thromboembolic complications of AF can lead to significant disabling consequences for patients, including transient ischemic attack, stroke, and pulmonary embolism. Early recognition of AF in general practice can lead to early intervention with managing the risks of these complications.

Current guidelines on the management of AF by the National Institute for Health and Care Excellence (NICE) advise identifying and managing the underlying causes of AF, treating the arrhythmia, and assessing and managing the risk of stroke in these patients [2]. Risk of stroke is determined by the CHA_2_DS_2_-VASc (Congestive heart failure, Hypertension, Age, Diabetes, previous Stroke/transient ischemic attack–VAScular disease) assessment tool [3], which guides the practitioner on commencing anticoagulation treatment to manage this risk. The risk of starting a patient on anticoagulation is assessed using the HAS-BLED (Hypertension, Abnormal renal/liver function, Stroke, Bleeding history or predisposition, Labile international normalized ratio, Elderly, Drugs/alcohol concomitantly) score, assessing for major bleeding risks [4].

Anticoagulation therapy aims to reduce the risk of thromboembolic events. This has been achieved using warfarin (a vitamin K antagonist) for many years. The introduction of direct oral anticoagulants (DOACs) such as apixaban and rivaroxaban to clinical practice has changed how AF is managed in clinical practice [5]. In comparison to warfarin, DOACs are observed to have similar or better mortality and vascular outcomes [6]. They have the added benefit of requiring no monitoring, as opposed to warfarin use [7]. Though studies have highlighted that increased number of patients in clinical practice are receiving treatment for AF [8], whether they are managed according to national or local guidelines is unclear. Better understanding of current management trends of AF in population will guide practitioners on how best to employ interventions in clinical practice to increase awareness of AF and early recognition.

The aim of this study is to understand the disease and prescribing trends related to AF in general practice. We also aim to develop a system to monitor practice-level indicators for AF of the same trends and evaluate the usability of this system. The primary objective of this study is to investigate if people with newly diagnosed AF treated with DOACs versus warfarin have any difference in stroke or all-cause mortality in long-term follow-up. The secondary objectives of this study are to develop an AF dashboard as an intervention method at a practice level and to evaluate the usability of the AF dashboard.

## Methods

### Study Design

This study combines qualitative and quantitative methods and therefore, was planned as a mixed method study [9-11]. It was run within the Oxford Royal College of General Practitioners Research and Surveillance Centre (RCGP RSC) sentinel network of general practices. The mixed method study was conducted in 2 phases. Phase 1 was a quantitative analysis of AF data. We conducted a repeated cross-sectional study to identify those patients diagnosed with AF during 2009-2019 and identified current therapeutic management of their AF. Phase 2 involved development and evaluation of a dashboard for monitoring data quality for AF. The dashboard provides the general practices in the RCGP RSC sentinel network an access to quality indicators related to AF management that are updated weekly.

### Phase 1: Quantitative Analysis of AF Data

#### Data Source

The initial phase involved a repeated cross-sectional retrospective analysis of data extracted from the RCGP RSC database, a surveillance network, which collects data from computerized medical records (CMR) from more than 3.5 million patients and over 300 general practices in England. CMRs are used during consultations to record patient conditions and prescriptions [12,13]. Anonymized clinical coded data from participating general practices were uploaded weekly to a secure network at the Clinical Informatics Research Group [12,14]. This study used NICE guidelines as the standard to compare the management of AF across this database.

#### Case Ascertainment

We used Read codes and CTV3 codes used in primary care CMR systems to identify patients with AF. Pay-for-performance (P4P) for chronic disease management has improved data quality related to AF in the recent past [15]. We excluded patients who had a previous stroke and those not on British National Formulary–recommended dose of DOAC. We included patients over 18 years with code for the diagnosis of AF who had been registered at any point between 2008 and 2019.

#### Exposures and Outcomes

We considered the exposure as continuous anticoagulant prescription for the first time after receiving an AF diagnosis. Although almost all anticoagulant prescriptions in the United Kingdom are issued from primary care if an anticoagulant is started in a hospital, we measured exposure from the first general practice prescription. We excluded patients who had an interval of greater than 90 days between anticoagulant prescriptions.

Outcomes are first records of stroke and all-cause mortality based on previously published Read codes [16,17] Stroke was included regardless of etiology, an approach used in other studies [18,19]. Study participants were followed for stroke and all-cause mortality till the end of the study period.

#### Covariates

We included variables likely to be used as indicators in prescribing anticoagulants: age-band, gender, and deprivation reporting Index of Multiple Deprivation (IMD) quintile. IMD is a national measure of socioeconomic status, which can be derived at an individual level from first part of postcode. We adjusted for comorbidities from that part of the stroke risk score (CHA_2_DS_2_-VASc), namely, heart failure, hypertension, stroke or transient ischemic attack, and myocardial infarction or peripheral vascular disease at baseline. We also included smoking status as a covariate.

### Statistical Analysis

Analysis of data extracted from general practice CMR was carried out using the statistical software R. We studied the influence of the anticoagulation regimen by evaluating the cause-specific hazard ratio and the subdistribution hazard ratios of both events. Additionally, we estimated cumulative incidence for both events by direct regression, utilizing the inverse of the probability of censoring weights method [15] with time-varying effects [20]. We will report and interpret both cause-specific and subdistribution analyses. The cause-specific hazard ratio is often interpreted as estimating etiological association, estimating associations between covariates, and the rate at which events occur in those participants who are event-free. The cause‐specific hazard ratio can be interpreted as a rate ratio. Whenever the proportional hazards assumption was violated, we interpreted all hazard ratios as time-averaged effects.

We will determine the (adjusted) incidence and prevalence of AF for each year along with the proportion of incident and prevalent cases treated with nothing, antiplatelet medications only (eg, aspirin), and anticoagulant therapy (warfarin/types of DOACs). We will also determine the number of strokes, transient ischemic attacks, deaths, and bleeds in the incident and prevalent cases per year.

### Phase 2: Development of a Dashboard for Monitoring AF

We developed an online dashboard for the general practices to understand the quality of their data across a variety of AF-related indicators. The AF dashboard provided 4 categories of information: (1) case ascertainment—incidence, prevalence, standardized prevalence, and any indicators related to P4P prevalence; (2) indications for therapy and risk factors; (3) management choices; and (4) quality. The dashboard was developed using Tableau data visualization software (Salesforce Inc). The initial dashboard is hosted on the public dashboard cloud server and accesses a publicly accessible database server (located within information technology infrastructure of the University of Oxford), which hosts only aggregated data to comply with the requirements of information governance. The dashboard allowed users to view the AF indicators only from their own practice.

We used the Think Aloud method to validate the usability of the AF dashboard. During usability experiment, study participants were instructed to verbalize their thoughts while concurrently conducting predefined tasks on the dashboard [21]. The design of the Think Aloud experiment involved participants engaging in 5 tasks. Participants were asked to verbalize their thought process while engaging in each of the tasks [22]. During the initial 4 tasks, the participants interacted with the 4 main sections of the dashboard. They observed the given information and interpreted it with respect to that particular aspect of AF management in their practice. As a fifth task, participants considered the overall dashboard and described the overall state of AF management in their practice in comparison to the RCGP RSC sentinel network.

#### Participants

We invited staff from general practices to cover a range of roles from primary care, including general practitioners, nurses, and practice managers. We provided remote participation for those who could not attend in person. We aimed to invite staff from practices participating in the RCGP RSC sentinel network. Our target sample size was about 30 participants based on the guidelines from previous studies [23].

#### Data Capture and Analysis

We recorded participant feedback and screen activity using GoToMeeting screen sharing software, version 10.5 (LogMeIn Inc). The audio component of the recordings was exported and transcribed by a professional transcription service. The transcripts were analyzed using NVivo, version 12 (QSR International), a qualitative analysis software. We followed a 3-step approach to analyze the transcripts.

1. *Mapping verbalized tasks to sections:* Each verbalized task description was extracted and mapped to the corresponding section of the dashboard. A “verbalized task” is the narrative description provided by a participant when interacting with particular component in the dashboard. We organized the verbalized tasks according to the template given in (Table S1 in Multimedia Appendix 1). The verbalized tasks were mapped to the dashboard sections, and similar feedbacks were grouped.
2. *Mapping verbalized tasks to usability problem classes:* For each section, we mapped the verbalized tasks to matching usability problem classes. We adapted the usability problem classification method used by Peute et al [21] and identified occurrences for each usability problem class (Table S2 in Multimedia Appendix 1). We also classified the identified verbalized tasks based on whether they were positive feedback, negative feedback, or suggestions for new features.
3. *Summarizing usability issues across sections/usability problem class:* The completed templates were analyzed to determine the critical sections and key usability problem classes that required attention.

### Ethical Considerations

For phase 1, approval was granted by the research committee of the RCGP RSC. The study did not meet the requirements for a formal ethics board review as per the NHS Health Research Authority’s research decision tool [24]. The study was conducted in line with the REporting of studies Conducted using Observational Routinely-collected Data (RECORD) guidelines [25]. For phase 2, personal data were not collected from the study participants. Participants were informed that their verbal responses and screen activities would be recorded. All participants received oral and written information about the study.

### Dissemination

The protocol and data produced from this work will be submitted for publication in a peer-reviewed journal covering the domains of cardiology, epidemiology, and primary care. Additional opportunities include presentation at seminars, conferences, and meetings.

## Results

We received funding for both phases in January 2019 and received approval from RCGP RSC research committee in March 2019. We completed data extraction for phase 1 in May 2019 and completed analysis in December 2019. We completed building the AF dashboard in May 2019. We started recruiting participants for phase 1 in May 2019 and concluded data collection in July 2019. We completed data analysis for phase 2 to in October 2019. As of October 2020, the publications reporting the results are under review.

## Discussion

The RCGP RSC sentinel network has a large dataset, which is representative of information regarding primary care practices on management of AF. The quantitative analysis will report on the current trends of anticoagulant prescribing using a real-world data set in the United Kingdom and the overall population effect of the use of DOAC use.

Missing/incomplete data could be a potential issue in this analysis. We intend to reduce the effect of this issue by incorporating an ontological approach, considering practice level variations in the anticoagulation prescribing.

The qualitative analysis of the dashboard will indicate the priority of sections that require an improved user experience. We intend to enhance the dashboard using the outcomes of the usability study and deploy to general practices in the RCGP RSC sentinel network.

As the incidence of AF has increased over the years, there is a greater focus on early recognition of AF in general practice to allow for early intervention. This study will give insights into the current trends in management of AF using real-world data from the Oxford RCGP RSC database. We anticipate that the outcomes of this study will be used to guide the development and implementation of an audit-based intervention tool for use in primary care settings to assist practitioners in identifying and managing AF.

## Appendix

Multimedia Appendix 1 Supplementary file with Table 1 and Table 2 described in the study design of phase 2.

## Abbreviations

AF: atrial fibrillation
CHA2DS2-VASc: Congestive heart failure, Hypertension, Age, Diabetes, previous Stroke/transient ischemic attack–VAScular disease
CMR: computerized medical records
DOAC: direct oral anticoagulant
HAS-BLED: Hypertension, Abnormal renal/liver function, Stroke, Bleeding history or predisposition, Labile international normalized ratio, Elderly, Drugs/alcohol concomitantly
IMD: Index of Multiple Deprivation
NICE: National Institute for Health and Care Excellence
P4P: pay-for-performance
RCGP RSC: Royal College of General Practitioners Research and Surveillance Center
RECORD: REporting of studies Conducted using Observational Routinely-collected Data
