# Supplementary material for: Improving the Management of Atrial Fibrillation in General Practice: Protocol for a Mixed Methods Study
Source: JMIR Res Protoc. 2020 Nov 9;9(11):e21259. doi: 10.2196/21259 (PMC7683254; doi:10.2196/21259)
Supplement: Multimedia Appendix 1 [file resprot_v9i11e21259_app1.docx]

# Improving the management of Atrial Fibrillation in General Practice: Protocol for a Mixed Method Study

## Supplementary file 1

Table S1. Task verbalization summary template for each participant

| **Section** | **Verbalized tasks** | **Positive** | **Negative** | **New features** |
| --- | --- | --- | --- | --- |
| 1. Case ascertainment | <<participant response>> |  |  |  |
| 1. Indications for therapy and risk factors | <<participant response>> |  |  |  |
| 1. Management choices | <<participant response>> |  |  |  |
| 1. Quality | <<participant response>> |  |  |  |
| 1. Overall AF management for practices | <<participant response>> |  |  |  |

Table S2. Template to map verbalized tasks to usability problem classes

| **Section** | **<<section name>>** | | |
| --- | --- | --- | --- |
| **Usability problem classification** | **Verbalized tasks** | | |
|  | **Positive** | **Negative** | **New features** |
| 1. Visibility of system status |  |  |  |
| 2. Error messages/ help instructions |  |  |  |
| 3. Meaning of labels/graphs |  |  |  |
| 4. Layout/ screen organization |  |  |  |
| 5. Dashboard controls |  |  |  |
